# DNA methylation intratumor heterogeneity in localized lung adenocarcinomas

**DOI:** 10.18632/oncotarget.15777

**Published:** 2017-02-28

**Authors:** Kelly Quek, Jun Li, Marcos Estecio, Jiexin Zhang, Junya Fujimoto, Emily Roarty, Latasha Little, Chi-Wan Chow, Xingzhi Song, Carmen Behrens, Taiping Chen, William N. William, Stephen Swisher, John Heymach, Ignacio Wistuba, Jianhua Zhang, Andrew Futreal, Jianjun Zhang

**Affiliations:** ^1^ Department of Thoracic/Head and Neck Medical Oncology, The University of Texas, MD Anderson Cancer Center, Houston, TX 77030, USA; ^2^ Department of Genomic Medicine, The University of Texas, MD Anderson Cancer Center, Houston, TX 77030, USA; ^3^ Department of Epigenetics and Molecular Carcinogenesis, The University of Texas, MD Anderson Cancer Center, Houston, TX 77030, USA; ^4^ Department of Bioinformatics and Computational Biology, The University of Texas, MD Anderson Cancer Center, Houston, TX 77030, USA; ^5^ Department of Translational Molecular Pathology, The University of Texas, MD Anderson Cancer Center, Houston, TX 77030, USA; ^6^ Department of Thoracic Surgery, The University of Texas, MD Anderson Cancer Center, Houston, TX 77030, USA; ^7^ Department of Cancer Biology, The University of Texas, MD Anderson Cancer Center, Houston, TX 77030, USA

**Keywords:** intra-tumor heterogeneity, non-small cell lung cancer, DNA methylation

## Abstract

Cancers are composed of cells with distinct molecular and phenotypic features within a given tumor, a phenomenon termed intratumor heterogeneity (ITH). Previously, we have demonstrated genomic ITH in localized lung adenocarcinomas; however, the nature of methylation ITH in lung cancers has not been well investigated. In this study, we generated methylation profiles of 48 spatially separated tumor regions from 11 localized lung adenocarcinomas and their matched normal lung tissues using Illumina Infinium Human Methylation 450K BeadChip array. We observed methylation ITH within the same tumors, but to a much less extent compared to inter-individual heterogeneity. On average, 25% of all differentially methylated probes compared to matched normal lung tissues were shared by all regions from the same tumors. This is in contrast to somatic mutations, of which approximately 77% were shared events amongst all regions of individual tumors, suggesting that while the majority of somatic mutations were early clonal events, the tumor-specific DNA methylation might be associated with later branched evolution of these 11 tumors. Furthermore, our data showed that a higher extent of DNA methylation ITH was associated with larger tumor size (average Euclidean distance of 35.64 (> 3cm, median size) versus 27.24 (<= 3cm), p = 0.014), advanced age (average Euclidean distance of 34.95 (above 65) verse 28.06 (below 65), p = 0.046) and increased risk of postsurgical recurrence (average Euclidean distance of 35.65 (relapsed patients) versus 29.03 (patients without relapsed), p = 0.039).

## INTRODUCTION

Cancer is a genetically heterogeneous disease. Cancer cells harbor distinct molecular and phenotypic features within a given tumor, a phenomenon termed intratumor heterogeneity (ITH) [18]. Genomic ITH has been found in numerous cancer types such as chronic lymphocytic leukemia, clear cell renal cell carcinoma, glioma, pancreatic, prostate, colorectal and lung cancers [1, 5, 7, 9, 12, 21, 23, 25]. ITH may have important clinical implications such as sampling bias from a single tumor biopsy, development of therapeutic resistance and disease recurrence [8, 25]. In addition to genomic aberrations, somatic epigenetic alterations may also impact neoplastic transformation and fitness [2, 3]. DNA methylation is a major component of epigenetic modification of the genome and predominantly occurs at cytosine residues within CpG dinucleotides (CpG sites) and clusters of CpG sites are termed “CpG islands” [11]. The pattern of DNA methylation in any given cell is a result of a dynamic process of methylation and demethylation. Once established, these patterns can be inherited without significant change from one cell generation to the next [20]. Altered DNA methylation is often observed in cancers with genome-wide DNA hypomethylation and site-specific hypermethylation of CpG islands [2, 6, 17].

Previously, we have characterized genomic ITH in 11 localized lung adenocarcinomas using a multi-region sequencing approach [25]. However, the nature of methylation ITH in lung cancer has yet to be fully explored. Here, we generated methylation profiles of the same multi-region DNA samples from the 11 localized lung adenocarcinomas and matched normal lung tissues used in our previous study [25]. DNA methylation status was evaluated utilizing the Illumina Infinium Human Methylation 450K BeadChip array covering more than 450,000 CpG sites and 99% RefSeq genes. We first performed unsupervised clustering analysis to investigate the variation of DNA methylation patterns within the tumors and across different tumors. To understand methylation changes during cancer evolution of these tumors, we defined the methylation status of each probe in tumor samples compared to matched normal lung tissues and categorized differentially methylated probes into early (i.e. clonal) and later (i.e. subclonal) events. We then examined genome-wide relationship between methylation and genomic changes. Lastly, we attempted to assess the association between DNA methylation profiles and clinicopathological features of these tumors.

## RESULTS

### Identification of DNA methylation intra- and inter-tumor heterogeneity

**Figure 1:**
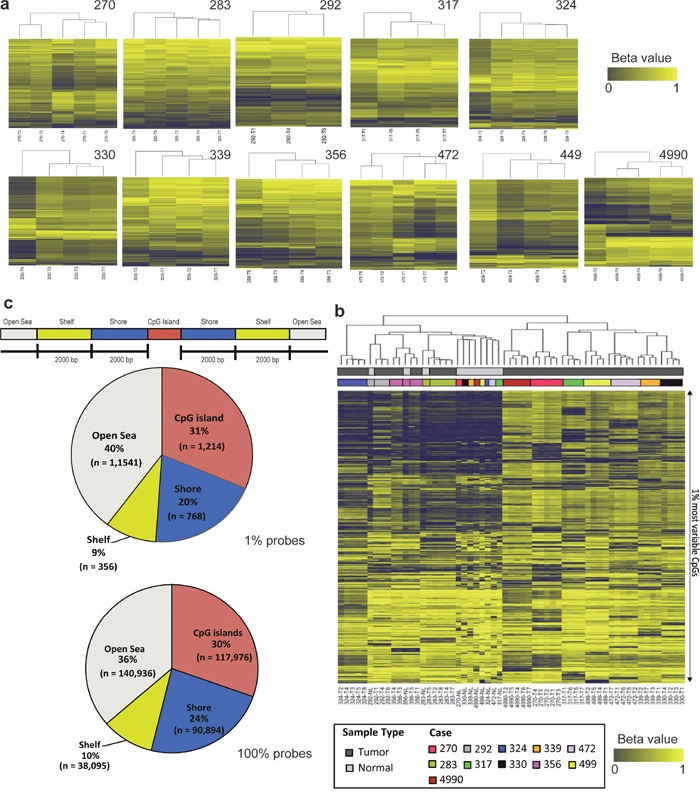
Assessment of methylation intratumor and intertumor heterogeneity of localized lung adenocarcinomas (**a**) Unsupervised hierarchical clustering of intratumoral DNA methylation. Columns are the tumor regions and rows are the DNA methylation status (beta values; ranged from 0 to 1) for the top 1% CpG probes (n = 4, 855). Dark blue denotes low and yellow indicates high methylation level. (**b**) Unsupervised hierarchical clustering of intertumoral DNA methylation across the cohort of 11 patients for the top 1% CpG probes (n = 3, 879). (**c**) Top: DNA methylation CpG probes are mapped to gene regions relatively to the proximity to CpG island. Bottom: Genomic distributions of the CpG probes obtained from 1% probes and 100% probes.

Genome-scale DNA methylation profiles were generated for the same cohort of 11 localized lung adenocarcinomas and matched normal lung tissues (Supplementary Table 1). Of these 11 patients, four have had disease relapse and the remaining patients are currently relapse-free. To determine the variation of DNA methylation patterns between different regions of the individual tumors and across the different tumors, we performed unsupervised hierarchical clustering of the most variable CpG probes. As shown in Figure 1a and Supplementary Figure 1a, heterogeneity was observed between different tumor regions within the same tumor; however, individual tumor regions were more similar to each other as compared to the matched normal lung tissues. When comparing all the samples across different patients, the normal lung tissues from different patients tended to cluster together while different tumor regions from the same patient always clustered together (Figure 1b and Supplementary Figure 1b). Taken together, these results demonstrated marked inter-individual methylation patterns and significant divergence between normal and tumor tissues. Next, we categorized the probes based on genomic locations by mapping the probes to their relative distance to CpG island and gene body [10]. Among the top 1% most variable probes across all regions of tumors, 40% were located in open sea, 31% were within a CpG island, 20% were in shores and 9% were in shelves (Figure 1c), which was similar to the genomic distribution of all probes.

### Tumor-specific methylation aberrations during cancer development

To understand methylation changes during cancer development for these 11 localized lung adenocarcinomas, we compared all tumor tissues to matched normal lung tissues and defined the methylation status of each probe in tumor samples as differentially methylated (i.e. beta value of tumor sample minus beta value of matched normal lung sample; Δ beta was > 0.3 or < -0.3 in at least one tumor region of a tumor) or unchanged. Among the differentially methylated probes, it was defined as clonal if Δ beta > 0.3 in all tumor regions of a given tumor or Δ beta < -0.3 in all tumor regions of a given tumor. Otherwise, it was defined as subclonal. On average 25% of all differentially methylated probes were shared by all regions from the same tumors (Table 1). These findings were in contrast to genomic ITH of these tumors; where on average 77% of all somatic mutations [25] were shared events amongst all regions of individual tumors (p = 5.821e-07, Student’s t test) (Table 1). Using a less stringent cutoff of Δ beta was > 0.2 or < -0.2, the difference between methylation and genomic ITH remained significant (p = 1.639e-05, Student’s t test) (Supplementary Table 2). Among the tumor suppressor genes that have been reported to be down-regulated by hypermethylation during cancer development [16], we observed an increased DNA methylation level (i.e. beta values > 0.3 when comparing to matched normal lung tissue) near the promoter regions (i.e. mapping to transcription start site from -500 to 200bp) of *SFRP1, RASSF1, GATA5, ESR1, RARB, CDKN2A, SFRP5, GATA4, SFRP2* and 34.3% (12 of 35) of these tumor-specific methylation were shared by all regions of individual tumors (Supplementary Figure 2), suggesting that these were early clonal events during development of these tumors. On the contrary, 95% (20 of 21) of known cancer gene mutations [25] in these tumors were clonal events (p = 4.631e-06, Fisher's Exact Test).

**Table 1:** Comparison of clonal tumor-specific DNA methylation and clonal genomic mutations of 11 localized lung adenocarcinomas

Case	Proportion of clonal tumor-specific DNA methylation	Proportion of clonal genomic mutations^§^
270	0.291	0.533
283	0.112	0.862
292	0.457	0.934
317	0.348	0.986
324	0.481	0.878
330	0.130	0.671
339	0.246	0.711
356	0.024	0.571
472	0.205	0.743
499	0.135	0.955
4990	0.292	0.595
Average	0.247	0.767
p-value	5.821e-07 (differentially DNA methylation versus genomic mutation)	-

§ Proportion of clonal genomic mutations were derived from previous study [25].

### Relationship between methylation and genomic landscape

**Figure 2:**
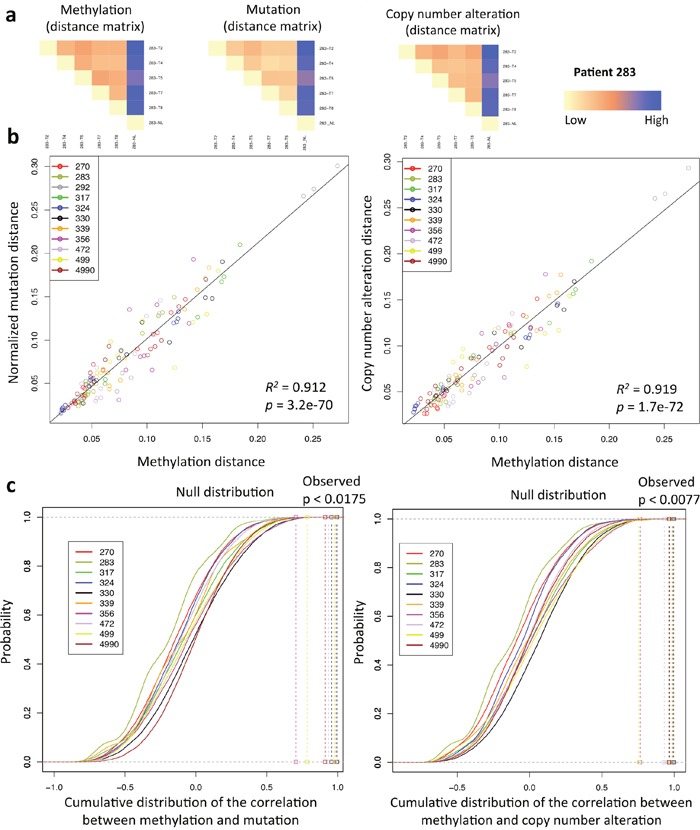
Relationship between methylation and genomic landscape (**a**) An illustration of methylation and genomic distance matrices comparison. Heat maps show the Euclidean distance for all samples of patient 283 based on methylation, mutation, and copy number alteration profiles. (**b**) Linear regression analysis of all samples between methylation and mutation or copy number alteration Euclidean distance matrices. With respect to the mutation data, each element of the resulting distance matrix was divided by the sum of mutation distance for each patient to obtain the normalized mutation distance. (**c**) Bootstrapping analysis of all samples. The correlation coefficient between methylation and mutation or copy number alteration Euclidean distance matrices of each patient was compared to the null distribution that was obtained by randomly shuffling the labels of methylation and genomic Euclidean distance matrices for 100,000 times.

We further investigated the relationship between methylation and genomic landscape of each tumor. To maximally utilize the data and capture the comprehensive genomic and methylation landscape of each sample, rather than using binary data, we calculated pairwise distances between each pair of samples from the same patient using beta values of all probes for methylation, allelic frequencies of all mutations and log2 ratios for copy number alterations (mutation and copy number data were obtained from previous study [25]). The comparison of distance matrices based on methylation, mutation and copy number changes displayed high similarity across all tumors (Figure 2a, Supplementary Figure 3). We then further measured this similarity by calculating the Pearson correlation coefficient between methylation and genomic distance matrices. A positive linear relationship was observed between methylation and mutation or copy number distances (*R^2^* = 0.912, *p* = 3.2e-70 for methylation versus mutation; *R^2^* = 0.919, *p* = 1.7e-72 for methylation versus copy number alterations, linear regression analysis) (Figure 2b, Supplementary Figure 4, Supplementary Figure 5). Subsequent bootstrapping analysis confirmed that the correlation was significant in all cases (p < 0.0175 for methylation versus mutation; p < 0.0077 for methylation versus copy number alterations) except for patient 292 who had only 3 tumor samples, which were insufficient for the analysis (Figure 2c). These data are consistent with the previous findings in prostate cancer and glioblastoma [4, 15] suggesting that the global landscapes of methylation and genomic were correlated to each another in these tumors.

To explore the potential mechanisms underlying the observed correlation between methylation and genomic landscape in this cohort, we first examined whether the methylation profiles were affected by copy number state or tumor purity and found no correlation between methylation status (i.e. beta values of array probes) and copy number state of corresponding chromosomal segments (i.e. log2 ratios) (r ranged from –0.0530 to 0.0352, Pearson correlation) or tumor purity in each sample (by pathologists review: r = 0.1444, p = 0.0963, Pearson correlation) (Supplementary Table 3). Then, we investigated whether mutations in genes directly regulating methylation [22] could be responsible for the correlation. However, we did not identify any detrimental mutation in these genes including *DNMT1, DNMT3B, IDH1, IDH2, TET1, TET2, TET3, UHRF1, EZH2*.

### Association between DNA methylation ITH and clinicopathological characteristics

With the full acknowledgement of small sample size in our cohort, we attempted to assess whether tumor-specific methylation change is associated with clinicopathological characteristics. We calculated the Euclidean distance between each tumor region to the matched normal lung tissue. The result showed that ever smokers (including current and former smokers) and larger tumors (> median size) tend to have a higher degree of overall tumor-specific methylation changes (average Euclidean distance of 90.47 for tumors > 3cm (median) versus 64.75 for tumors <= 3 cm, p=0.026; average Euclidean distance of 85.57 for tumors from ever smokers versus 60.68 for tumors from never smokers, p = 0.041, Student’s t-test (Supplementary Figure 6), while tumor size and smoking status are not correlated to each other (p = 0.256, Student’s t-test).

**Figure 3:**
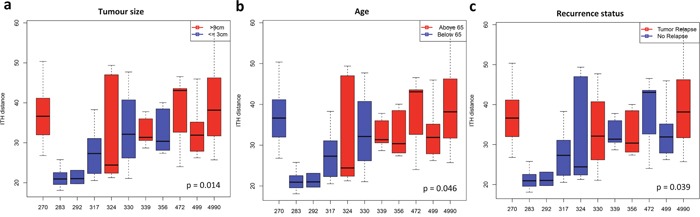
Association between DNA methylation ITH level and patient characteristics Boxplots show the methylation ITH as the Euclidean distance between different tumor regions within each tumor. Solid horizontal line within each box is the median; solid box shows the 25 and 75 percentile, and caps show the 5 and 95 percentile. (**a**) The association of methylation ITH and tumor size - average Euclidean distance 35.64 (> 3 cm, median) versus 27.24 (<= 3 cm). (**b**) The association of methylation ITH with advanced age - average Euclidean distance 34.95 (above 65) versus 28.06 (below 65). (**c**) The association of methylation ITH with recurrence status - average Euclidean distance 35.65 (relapsed patients) versus 29.03 (patients without relapsed). Matched normal lung tissues were excluded in this analysis. All p-values are from Student’s t-test.

We further quantified the extent of methylation ITH of each tumor using mean Euclidean distances between different tumor regions within the same tumors and assessed the association of DNA methylation ITH with patient characteristics (Supplementary Table 1). Our analysis demonstrated that a higher extent of DNA methylation ITH was associated with larger tumor size (35.64 (> 3cm) versus 27.24 (<= 3cm), p = 0.014, Student’s t-test) and advanced age (34.95 (above 65) verse 28.06 (below 65), p = 0.046, Student’s t-test) (Figure 3a and 3b). No association of DNA methylation ITH with gender or smoking status was observed (Supplementary Figure 7). Of particular interest, the four relapsed patients demonstrated a significantly higher level of methylation ITH than patients who have not relapsed (35.65 (relapsed) versus 29.03 (not relapsed), p = 0.039, Student’s t-test) (Figure 3c). The observed correlations appear to be independent from each other (tumor size versus relapsed, p = 0.8642, Student’s t-test; tumor size versus age, r = 0.256, p = 0.0654, linear regression analysis; age versus relapse, p = 0.7543, Student’s t-test). With the limited sample size, our data suggest that DNA methylation ITH might be associated with inferior clinical outcome in patients with localized lung adenocarcinomas and an ongoing study with a larger cohort is validating these interesting findings.

## DISCUSSION

In this study, we investigated the nature of methylation ITH in 11 localized lung adenocarcinomas using multiregional sampling approach. The DNA methylation array data revealed evidence of heterogeneity within the same tumors, but to a much less extent compared to inter-individual heterogeneity. Consistent with our observations, recent work by Brocks et al. showed that different regions of the same tumor were more similar to each other than those from different individuals [4].

Tumors are not only masses of malignant cells, but are a complex milieu consisting of many cell types, including epithelial cells, blood and lymphatic vessel endothelial cells, and infiltrating immune cells. Each cell type likely harbors a distinct methylation profile; therefore, the overall methylation heterogeneity may reflect not only the individual methylation profiles of distinct cancer cell populations, but also inform on the cell types and/or methylation status of each component. In the current study, all tumor samples had at least 40% viable cancer cells and 37 of 48 tumor samples had viable cancer cells of 50% or more (Supplementary Table 3), while the proportion of non-epithelial cell components such as fibroblasts or immune cells was small in the majority of tumors. Furthermore, different tumor regions from the same patient always clustered together despite their differences in tumor purity or proportion of non-epithelial cell components, while the normal lung tissues from different patients clustered together. In fact, patient 270 with uniformly high tumor purity in all tumor regions (80%, 85%, 85%, 90% and 100% viable malignant cells) had one of the highest methylation ITH, while patient 283 with relatively low tumor purity in individual tumor regions (40%, 50%, 60%, 75% and 80% viable malignant cells) demonstrated the most homogenous methylation patterns among different tumor regions (Figure 3 and Supplementary Figure 6). Taken together, our data suggest that DNA methylation intra- and inter-tumor heterogeneity may be mainly attributed to spatial difference in methylation of lung cancer cells.

Profiling ITH using multi-region sampling approach provides an opportunity to reconstruct the tumor’s evolution in cancer development. Previously, we characterized genomic ITH and found that on average 77% of all somatic mutations and 95% of known cancer gene mutations were clonal events. In the current study, only approximately 25% of all differentially methylated probes (compared to matched normal lung tissues) were clonal events shared by all regions of individual tumors. With the limited sample size, these findings suggest the possibility that while the majority of somatic mutations are early molecular events during cancer development, the tumor-specific methylation may be associated with later branched evolution in these 11 tumors.

Although the tumor-specific methylation (compared to matched normal lung tissues) and somatic genomic mutations appear to occur at different molecular times during cancer development in these 11 localized lung adenocarcinomas, the comprehensive DNA methylation landscape was significantly correlated with genomic landscape in these tumors. Recent studies have also shown that the patterns of methylation and genomic landscapes were highly correlated in prostate and brain cancers [4, 15]. However, the mechanisms underlying this correlation are still unknown.

Although the sample size in this study is small, our data demonstrated that the extent of methylation ITH might be associated with larger tumor size (average Euclidean distance of 35.64 (> 3cm, median size) versus 27.24 (<= 3cm), p = 0.014, Student’s t-test), advanced age (average Euclidean distance of 34.95 (above 65) verse 28.06 (below 65), p = 0.046, Student’s t-test) and increased risk of postsurgical recurrence (average Euclidean distance of 35.65 (relapsed patients) versus 29.03 (patients without relapsed), p = 0.039, Student’s t-test) in lung cancers. Similar findings were also observed in chronic lymphocytic leukemia where a high level of methylation heterogeneity was associated with adverse clinical outcome [13]. These data suggest that methylation ITH may have both biological and clinical impact. Studies with a larger cohort are warranted to validate these intriguing findings and explore the potential of methylation ITH as a prognostic biomarker.

## MATERIALS AND METHODS

### Patient material, sample collection and processing

All 11 patients’ history, method of collection and processing were previously described in Zhang et al. [25]. To date, patient 356 has developed recurrence. Please see Supplementary Table 1 for the updated clinical information.

### Illumina 450K DNA methylation

Genomic DNA (approximately 500 ng) was bisulfite converted using EZ DNA Methylation Kit (Zymo Research Corp. Irvine, CA, USA) following the manufacturer’s protocol. Bisulfite converted DNA materials were then processed and hybridized to the Infinium HumanMethylome 450k arrays (Illumina, San Diego, CA, USA) according to manufacturer’s recommendation.

Preprocessing and initial quality assessments of the raw data were examined using the following Bioconductor R packages. Subset-quantile within-array normalization (SWAN) [14] was used to normalize raw methylation values. IlluminaHumanMethylation450k.db annotation package was used to annotate the CpG probes location. Before any genomics and statistical analyses were conducted, we normalized and inspected the methylation data for the presence of substantial confounding batch effects. No obvious batch effect was observed.

### Clustering analysis

For inter-individual DNA methylation analysis, probes on sex chromosomes (Chr X or Y) and probes containing single-nucleotide polymorphism (dbSNP137 common) were filtered out to avoid potential confounding effects. The remaining 387,901 probes were used to calculate the Euclidean distance between different samples and unsupervised hierarchical clustering (complete linkage) was performed for the most variable probes across the cohort at different cut-offs (i.e. 1% = 3,879 CpGs; 2% = 7,758 CpGs; 5% = 19,395 CpGs; 10% = 38,790 CpGs).

For intratumoral DNA methylation analysis for each patient, filtering was not applied. A total of 485,512 probes were used for calculation of Euclidean distance and unsupervised hierarchical clustering (complete linkage) was performed for the top 1% probes (n = 4,855 CpGs).

### Correlation analysis

Methylation-based Euclidean distance matrices were generated based on beta values from all probes (n = 485,512). Mutation and copy number alteration profiles were derived from previous study [25]. Mutation-based Euclidean distance matrices were generated based on variant allele frequencies of all somatic mutations while the copy number alteration-based Euclidean distance matrices were based on log2 ratios (i.e. tumor DNA versus matched germline DNA), where the segmentation data was converted to a gene by sample matrix using Bioconductor R package ‘CNTools’ [24].

Pearson’s correlation coefficient was calculated to estimate the similarity between methylation and genomic distances matrices. A linear regression was used to determine the relationship between the similarities of methylation and mutation or copy number alteration Euclidean distances. Bootstrapping analysis:the null distribution for each patient was generated by randomly shuffling the labels of methylation and genomic Euclidean distance matrices for 100,000 times and then calculated the correlation coefficient of the resulting matrices for each bootstrap. An empirical p value was estimated by comparing the correlation coefficient between methylation and genomic Euclidean distance matrices of each patient to the null distribution.

### Statistical analysis

All statistical analyses were conducted using R environment for statistical computing and visualization [19].

## SUPPLEMENTARY MATERIALS FIGURES AND TABLE




