# Supplementary material for: DNA methylation intratumor heterogeneity in localized lung adenocarcinomas
Source: Oncotarget. 2017 Feb 28;8(13):21994–2002. doi: 10.18632/oncotarget.15777 (PMC5400640; doi:10.18632/oncotarget.15777)
Supplement: Supplementary file 1 [file oncotarget-08-21994-s001.pdf]

DNA methylation intratumor heterogeneity in localized lung adenocarcinomas

SUPPLEMENTARY MATERIALS

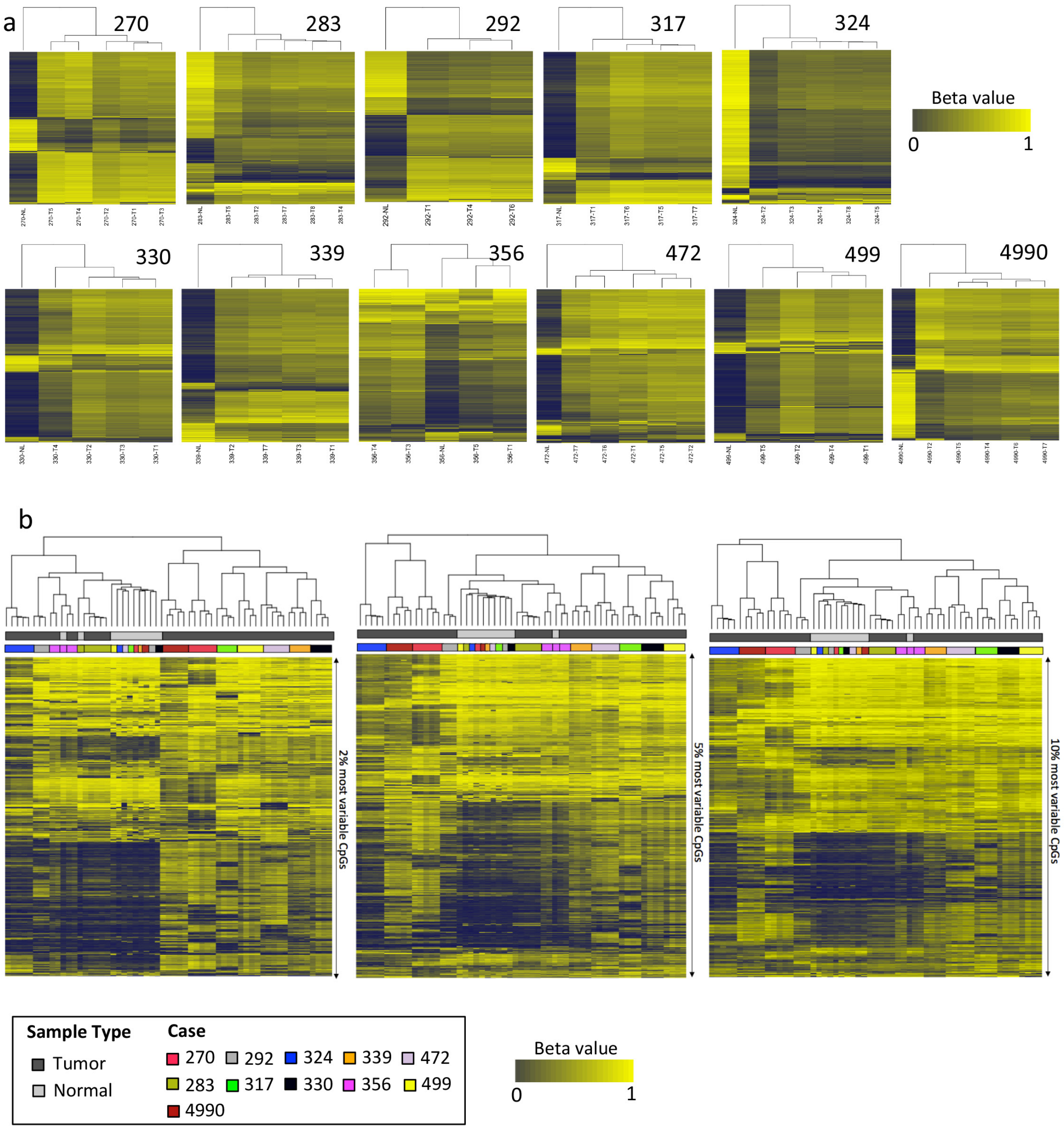

Supplementary Figure 1: Intra-tumoral and inter-tumoral DNA methylation patterns analysis. (a) Unsupervised hierarchical clustering of DNA methylation patterns of different regions within the same tumor at per patient level. Columns are the samples and rows are the DNA methylation status (beta values) of the top 1% CpG probes (n = 4, 855). The CpG probes location is depicted on the left column of the heat map. Blue denotes low and yellow indicates high methylation level (ranged from 0 to 1). **(b)** Unsupervised hierarchical clustering of DNA methylation patterns across all 11 patients using the most variable CpG at 2% (n = 7, 758), 5% (n = 19, 395), 10% (n = 38, 790) cutoffs.

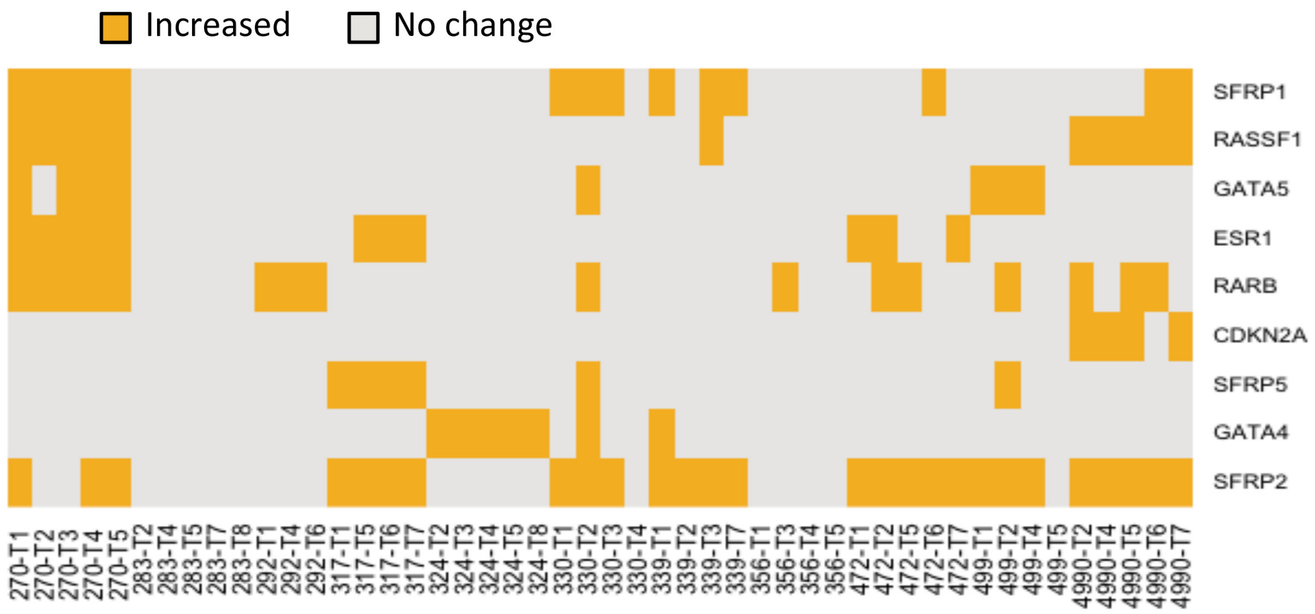

Supplementary Figure 2: Heat map showing increased DNA methylation of known cancer genes across different tumor regions. The methylation status of each gene near the promoter region was calculated as the average beta value of all the probes mapping to the transcription start site (-500 to 200 bp). Orange indicates increased DNA methylation level (i.e. average beta values > 0.3 over matched normal lung tissue). Grey denotes no change in DNA methylation level.

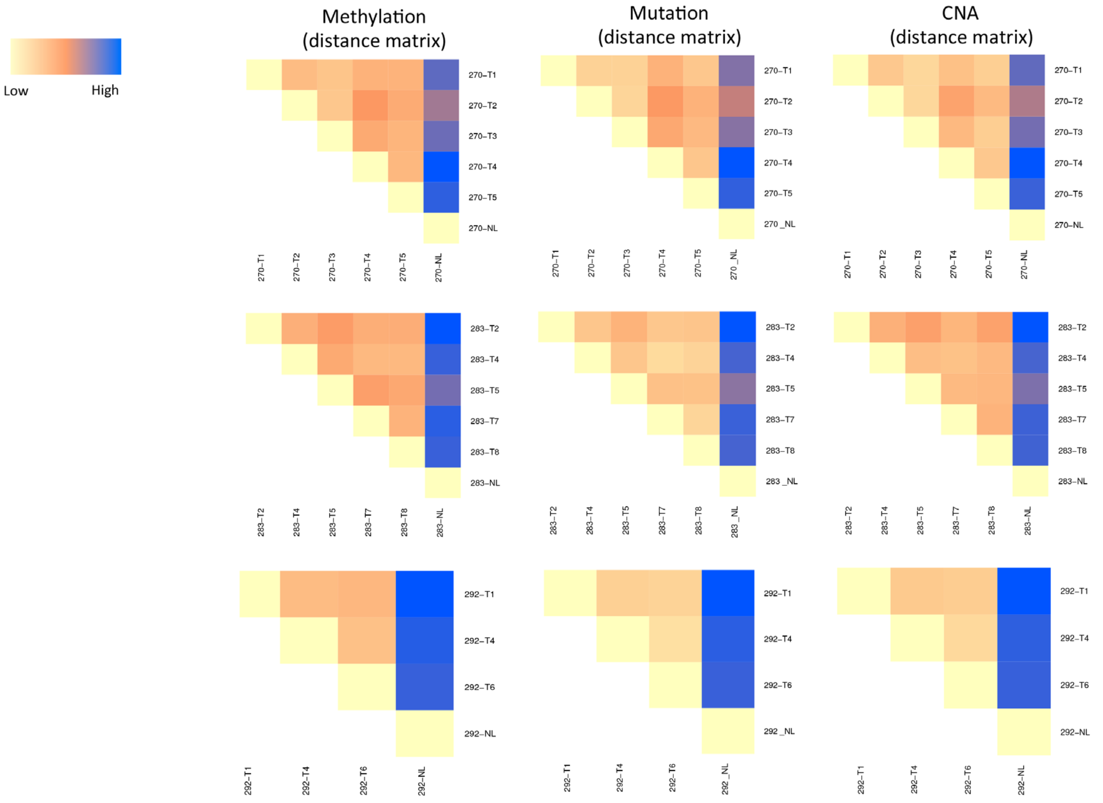
Low
High
Methylation
(distance matrix)
Mutation
(distance matrix)
CNA
(distance matrix)
270–T1
270–T2
270–T3
270–T4
270–T5
270–NL
283–T2
283–T4
283–T5
283–T7
283–T8
283–NL
292–T1
292–T4
292–T6
292–NL

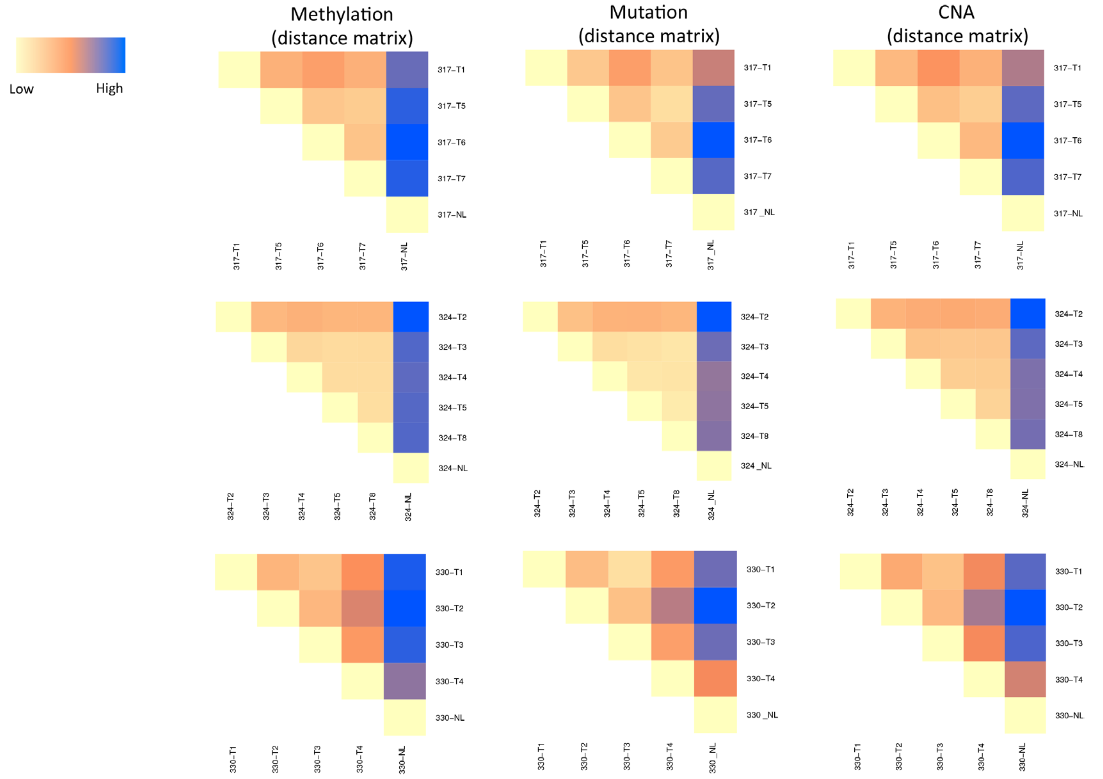
Low
High
Methylation
(distance matrix)
Mutation
(distance matrix)
CNA
(distance matrix)
317–T1
317–T5
317–T6
317–T7
317–NL
324–T2
324–T3
324–T4
324–T5
324–T8
324–NL
330–T1
330–T2
330–T3
330–T4
330–NL

(Continued)

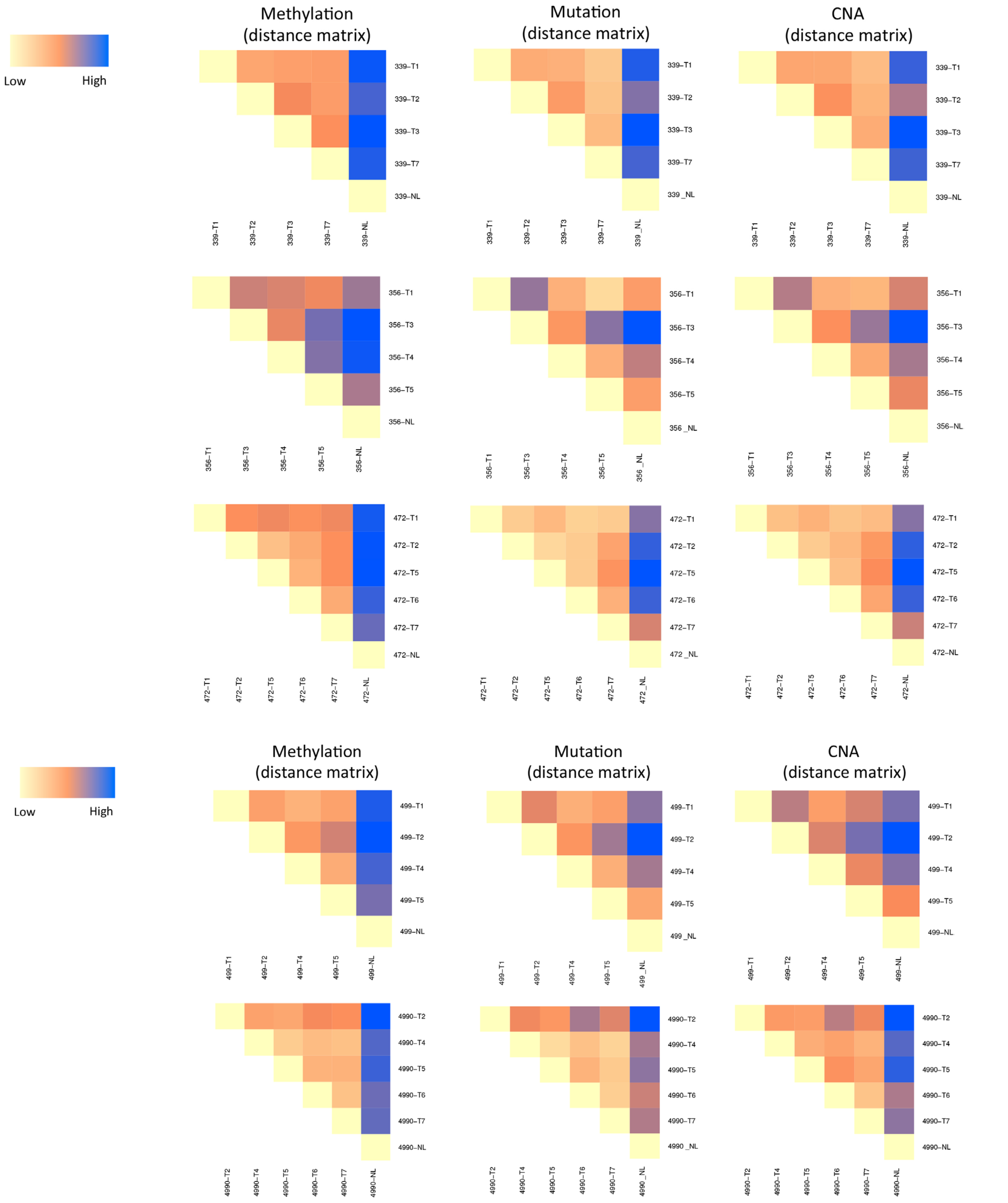

Supplementary Figure 3: Comparison between methylation and genomic distance matrices. Heat maps show the Euclidean distance for all samples of 11 patients based on methylation, copy number alteration and methylation patterns.

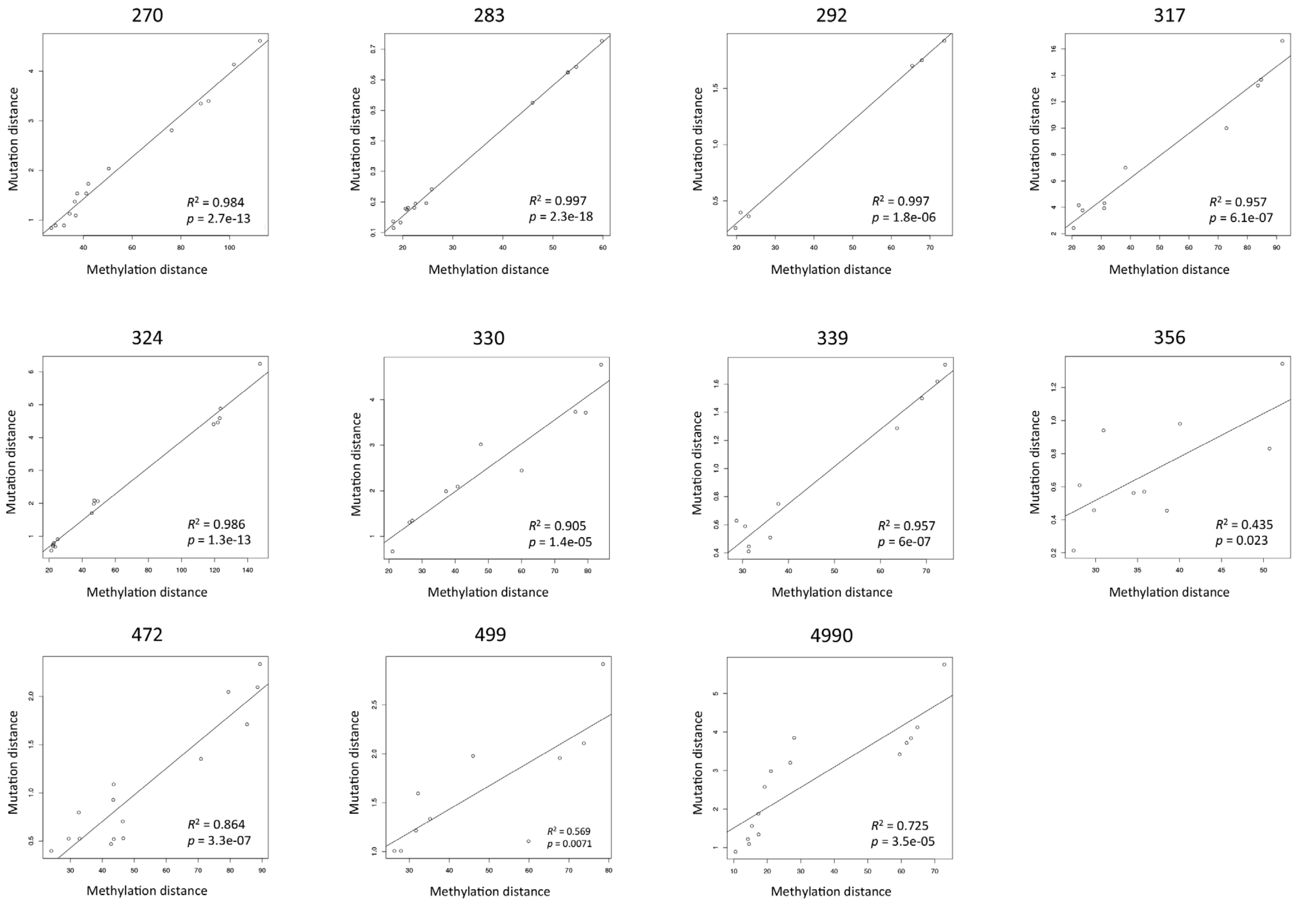

Supplementary Figure 4: Linear relationship between DNA methylation and mutation Euclidean distances for all 11 patients. X-axis indicates the values of Euclidean distance matrix based on DNA methylation profiles. Y-axis denotes the values of Euclidean distance matrix based on mutation (i.e. variant allele frequencies). The fitted line indicates the result of the linear regression analysis.

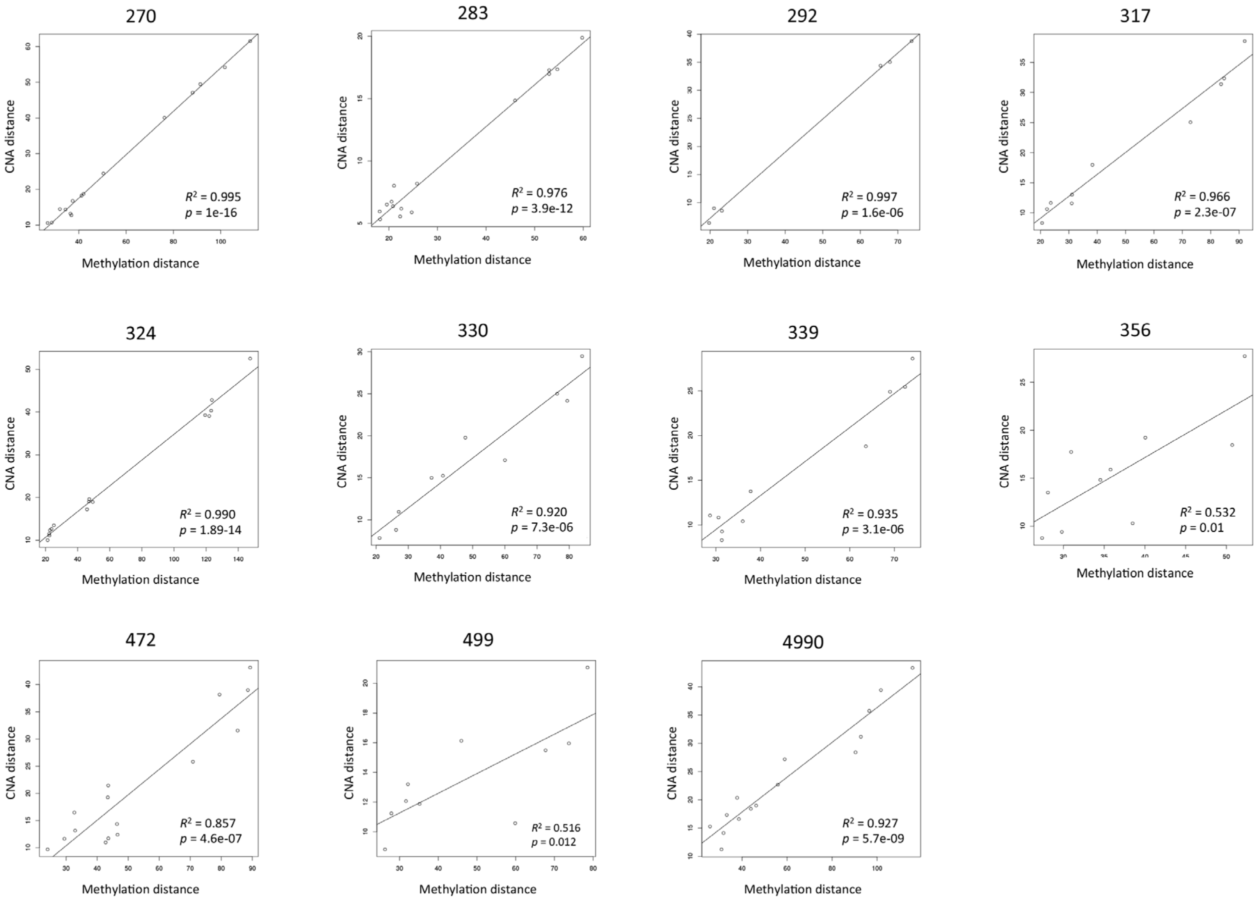

Supplementary Figure 5: Linear relationship between DNA methylation and copy number alteration Euclidean distances for all 11 patients. X-axis indicates the values of Euclidean distance matrix based on DNA methylation profiles. Y-axis denotes the values of Euclidean distance matrix based on copy number alteration (i.e. log2 ratios). The fitted line indicates the result of the linear regression analysis.

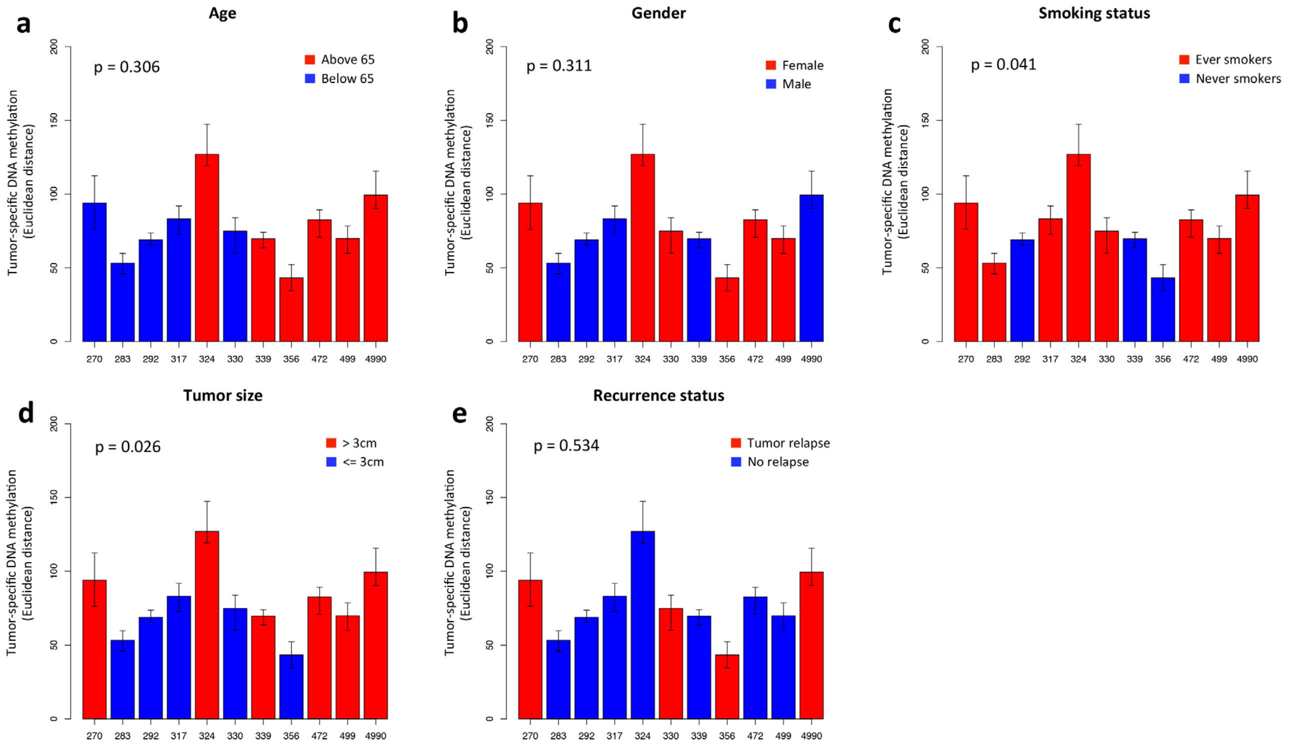

Supplementary Figure 6: Association between tumor-specific methylation and patient characteristics. Bar charts showing the tumor-specificmethylation level as the average Euclidean distance between different tumors regions within the same tumor and their matched normal lung tissues using all 485,512 probes. **(a)** The association between tumor-specific methylation and age - average Euclidean distance 82.03 (above 65) versus 74.88 (below 65). **(b)** The association between tumor-specific methylation and gender - average Euclidean distance 81.98 (female) versus 74.94 (male). **(c)** The association between tumor-specific methylation and smoking status - average Euclidean distance 85.57 (ever smokers) versus 60.68 (never smokers). **(d)** The association between tumor-specific methylation and tumor size - average Euclidean distance 90.47 (> 3 cm, median) versus 64.75 (<= 3 cm). **(e)** The association between tumor-specific methylation and recurrence status - average Euclidean distance 77.90 (relapsed patients) versus 79.28 (patients without relapsed). All p-values are from Student's *t* test.

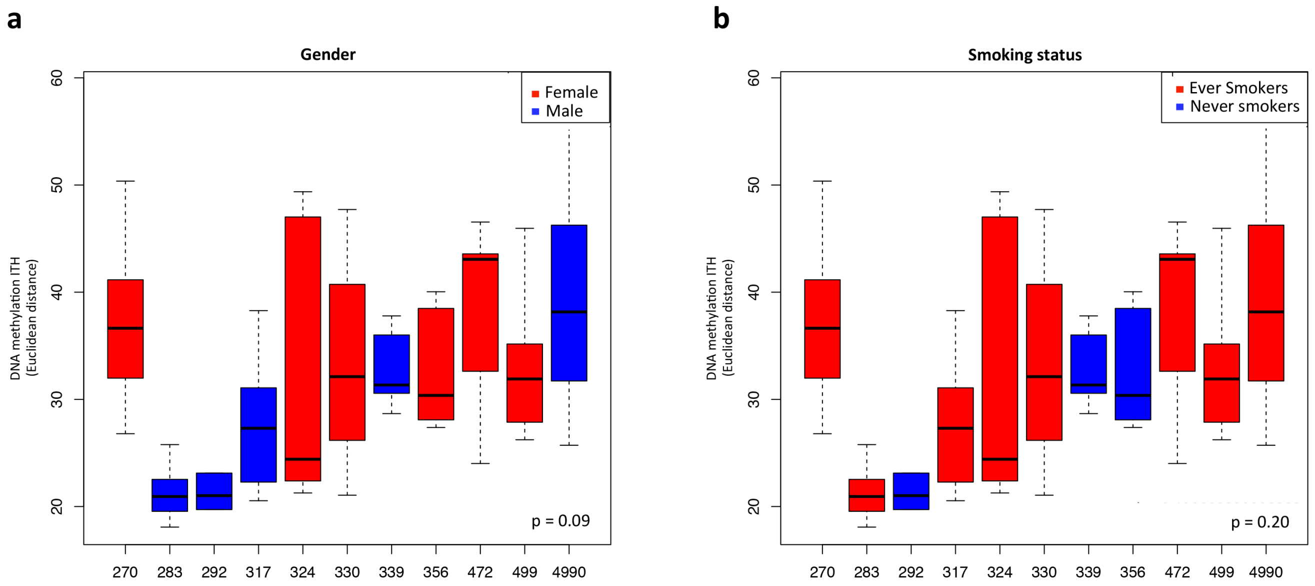

Supplementary Figure 7: Association between DNA methylation ITH level and patient characteristics. Boxplots show the DNA methylation ITH as the Euclidean distance between different tumor regions within each tumor using all 485,512 probes. Solid horizontal line within each box is the median; solid box shows the 25 and 75 percentile, and caps show the 5 and 95 percentile. **(a)** The association between methylation ITH and gender (average Euclidean distance 34.45 (female) versus 28.66 (male)). **(b)** The association between methylation ITH and smoking status (average Euclidean distance 32.96 (ever smokers) versus 28.78 (never smokers)). Matched normal lung tissues were excluded in this analysis. All p-values are from Student's t-test.

Supplementary Table 1: Patient characteristics

Case	Age/Gender	Smoking status	Tumor size (cm)	Pathological Node Stage	Adjuvant Therapy	Follow up (months)	Recurrence
270	62/F	Former: <5 pack3year, quit 20 years ago, continuous exposure to second3hand smoking	4.5	N1	Chemo	25	Yes
283	50/M	Former: 25 pack3year, quit 6 years ago	2	N2	Chemo followed by radiation	40	No
292	57/F	Never	3	N0	No	38	No
317	55/M	Former: 50 pack year, quit 5 years ago	2.8	N0	No	29	No
324	68/F	Current: 20 pack year, had cut down to 2 cigarettes a day at the time of cancer diagnosis.	3.5	N0	Radiation for positive margins	39	No
330	59/F	Former: 25 pack year, quit 2.5 years ago	2	N0	No	-	Yes
339	73/M	Never	3.2	N0	No	20	No
356	69/F	Never	2.2	N0	No	39	Yes
472	74/F	Former: 8 pack year, quit 42 years ago	3.3	N0	No	39	No
499	73/F	Former: 53 pack year, quit 5 years ago	4.5	N0	No	35	No
4990	75/M	Former: 32 pack year, quit 43 years ago	4.6	N1	No	13	Yes

Supplementary Table 2: Comparison of clonal tumor-specific DNA methylation and clonal genomic mutations

Case	Proportion of clonal tumor-specific DNA methylation[1]	Proportion of clonal genomic mutations[2]
270	0.389	0.533
283	0.255	0.862
292	0.589	0.934
317	0.494	0.986
324	0.616	0.878
330	0.263	0.671
339	0.320	0.711
356	0.039	0.571
472	0.311	0.743
499	0.263	0.955
4990	0.376	0.595
Average	0.356	0.767
p-value	1.639e-05 (differentially DNA methylation versus genomic mutation)	-

[1] Among all the differentially methylated probes (beta value of tumor sample minus beta value of matched normal lung; Δ beta > 0.2 or Δ beta < -0.2 in at least one tumor region of a given tumor), it was defined as clonal if Δ beta > 0.2 in all tumor regions of a given tumour or Δ beta < -0.2 in all tumor regions of a given tumor. Otherwise, it was defined as subclonal.
[2] Proportion of clonal genomic mutation derived from previous study (Zhang et al. 2014).

Supplementary Table 3: Tumor cell content

See Supplementary File 1
