# Supplementary material for: DNA methylation intratumor heterogeneity in localized lung adenocarcinomas
Source: Oncotarget. 2017 Feb 28;8(13):21994–2002. doi: 10.18632/oncotarget.15777 (PMC5400640; doi:10.18632/oncotarget.15777)
Supplement: Supplementary file 2 [file oncotarget-08-21994-s002.docx]

**Table S3 Tumor cell content**

| **Case** | **Regions collected** | **Composition** | | | |
| --- | --- | --- | --- | --- | --- |
|  |  | **% of viable malignant cells** | **% of fibrosis** | **% of necrosis** | **% of lymphoid cell infiltration** |
| 270 | 270-T1 | 85 | 0 | 0 | 5 |
|  | 270-T2 | 100 | 0 | 0 | 0 |
|  | 270-T3 | 80 | 5 | 0 | 5 |
|  | 270-T4 | 90 | 0 | 0 | 5 |
|  | 270-T5 | 85 | 0 | 0 | 5 |
| 283 | 283-T2 | 80 | 10 | 0 | 10 |
|  | 283-T4 | 75 | 10 | 0 | 10 |
|  | 283-T5 | 50 | 0 | 0 | 5 |
|  | 283-T7 | 60 | 20 | 0 | 10 |
|  | 283-T8 | 40 | 0 | 0 | 5 |
| 292 | 292-T1 | 45 | 0 | 0 | 5 |
|  | 292-T4 | 55 | 0 | 0 | 5 |
|  | 292-T6 | 45 | 0 | 0 | 5 |
| 317 | 317-T1 | 50 | 10 | 0 | 10 |
|  | 317-T5 | 55 | 5 | 0 | 10 |
|  | 317-T6 | 60 | 0 | 10 | 10 |
|  | 317-T7 | 55 | 10 | 10 | 5 |
| 324 | 324-T2 | 75 | 0 | 0 | 5 |
|  | 324-T3 | 60 | 0 | 10 | 20 |
|  | 324-T4 | 50 | 0 | 10 | 20 |
|  | 324-T5 | 60 | 0 | 0 | 20 |
|  | 324-T8 | 70 | 0 | 0 | 20 |
| 330 | 330-T1 | 90 | 0 | 0 | 10 |
|  | 330-T2 | 40 | 0 | 0 | 5 |
|  | 330-T3 | 60 | 10 | 0 | 10 |
|  | 330-T4 | 40 | 10 | 0 | 10 |
| 339 | 339-T1 | 50 | 20 | 0 | 10 |
|  | 339-T2 | 40 | 20 | 0 | 10 |
|  | 339-T3 | 75 | 10 | 0 | 5 |
|  | 339-T7 | 60 | 10 | 0 | 10 |
| 356 | 356-T1 | 65 | 10 | 0 | 5 |
|  | 356-T3 | 40 | 10 | 0 | 10 |
|  | 356-T4 | 65 | 10 | 0 | 5 |
|  | 356-T5 | 40 | 0 | 0 | 10 |
| 472 | 472-T1 | 60 | 0 | 0 | 10 |
|  | 472-T2 | 45 | 30 | 0 | 5 |
|  | 472-T5 | 75 | 10 | 0 | 5 |
|  | 472-T6 | 70 | 0 | 0 | 10 |
|  | 472-T7 | 55 | 0 | 0 | 15 |
| 499 | 499-T1 | 40 | 10 | 0 | 20 |
|  | 499-T2 | 40 | 20 | 0 | 10 |
|  | 499-T4 | 60 | 5 | 0 | 5 |
|  | 499-T5 | 70 | 5 | 0 | 5 |
| 4990 | 4990-T2 | 95 | 0 | 0 | 5 |
|  | 4990-T4 | 55 | 10 | 0 | 5 |
|  | 4990-T5 | 65 | 10 | 0 | 5 |
|  | 4990-T6 | 60 | 15 | 0 | 5 |
|  | 4990-T7 | 60 | 5 | 0 | 5 |
